# Venom ophthalmia (keratoconjunctivitis) caused by nuchal gland secretion of *Rhabdophis tigrinus*: case report

**DOI:** 10.1186/s12348-022-00308-z

**Published:** 2022-10-07

**Authors:** Kazuki Matsuura, Yoshitsugu Inoue

**Affiliations:** 1 Nojima Hospital, 2714-1, Sesaki-machi, Kurayoshi-city, Tottori 682-0863 Japan; 2 Tottori University, 36-1, Nishi-cho, Yonago-city, Tottori 683-8504 Japan

**Keywords:** *Rhabdophis tigrinus*, Venom ophthalmia, Bufadienolides, Keratoconjunctivitis

## Abstract

*Rhabdophis tigrinus* (*R. tigrinus*) is a common colubrid snake that possesses a series of paired sac-like nuchal glands behind the head. When pressure is applied to the nuchal area, the thin skin over the nuchal glands can rupture and release secretions. In Japan, 19 cases of ophthalmia caused by the nuchal gland secretion of *R. tigrinus* have been reported. However, only one case has been documented in an English report. A 72-year old woman was sprayed by the nuchal gland fluid of *R. tigrinus* in her right eye. She presented with symptoms of eye pain and blurred vision. A slit-lamp examination revealed diffuse superficial keratitis, corneal stromal edema with Descemet membrane folds, and conjunctival injection. The best-corrected visual acuity (BCVA) of her right eye was 0.6. She was prescribed 0.5% moxifloxacin and 0.1% fluorometholone eye drops four times a day, and the symptoms resolved without sequelae within 5 days. The BCVA in the right eye improved to 1.0. In previous reports, ophthalmic examinations revealed conjunctivitis, keratitis, and corneal edema with Descemet membrane folds. Topical antibiotics and corticosteroid were prescribed in most cases, and eyes healed within 5-7 days without any sequelae. While corneal edema may resolve spontaneously in a few days when inhibition of the toxin has ceased, the use of topical steroids is recommended, as it can increase the activity of Na/K pumps that remained functional, thereby accelerating recovery. In fact, our patient used a topical steroid and recovered without sequelae.

## Introduction

**Fig. 1:**
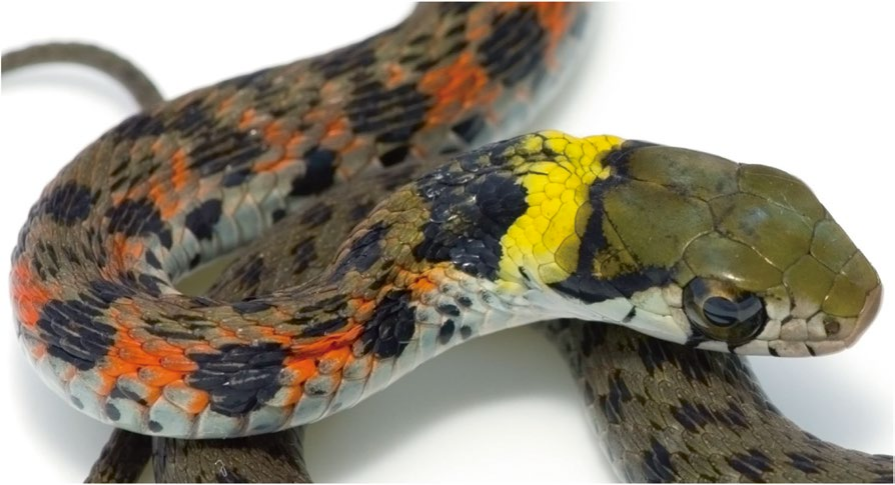
*Rhabdophis tigrinus*. A common colubrid snake found in eastern Asia. The average total length is usually 50–150 cm

*Rhabdophis tigrinus* (*R. tigrinus*), also known as tiger keelback or yamakagashi, is a common colubrid snake that is found in a wide geographical region, including southeastern Russia, northern and eastern China, Korea, Japan, Vietnam, and Taiwan [1]. Its dorsal color pattern is olive-drab green, with black and bright orange crossbars or spots from the neck to the body (Fig. 1).

*R. tigrinus* has two sets of poisonous glands—Duvernoy’s glands in the maxilla and nuchal glands in the dorsal skin of the neck. In Japan, the poison secreted by the nuchal gland of *R. tigrinus* causes ophthalmia when sprayed into the eyes [2, 3]. To the best of our knowledge, only one case has been documented in English literature so far [1]. Here, we report a case of ophthalmia caused by the nuchal gland secretion of *R. tigrinus*.

## Case presentation

**Fig. 2:**
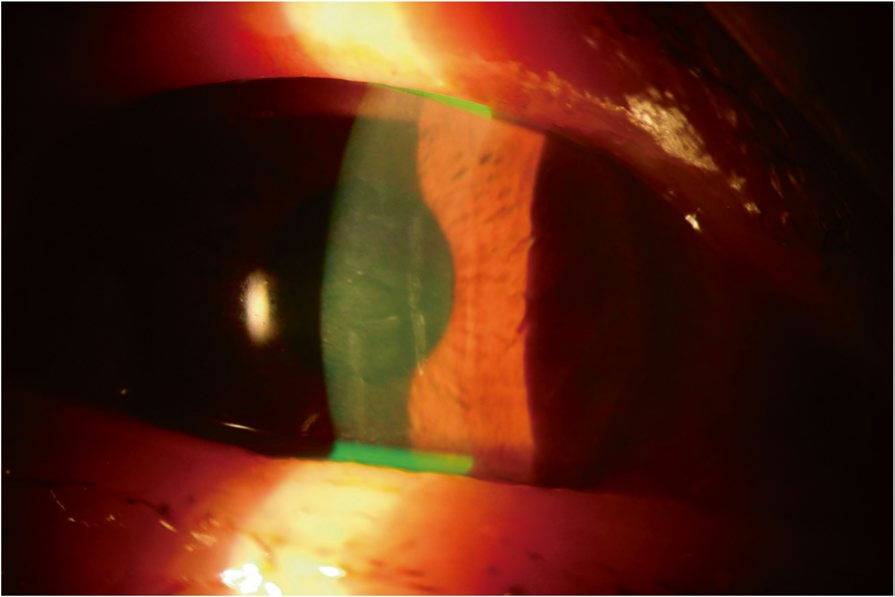
Slit-lamp examination revealed severe corneal edema with Descemet membrane folds

The patient was a 72-year-old woman whose right eye was sprayed by the nuchal gland fluid of *R. tigrinus* approximately 12 h before she visited our clinic. The eye had been rinsed with water immediately after coming into contact with the snake’s fluid. She presented with symptoms of foreign body sensation, eye pain, and blurred vision. Slit-lamp examination revealed diffuse superficial keratitis, corneal stromal edema with Descemet membrane folds, and conjunctival injection. Neither cell nor flare in the anterior chamber was detected, and pupil reaction was normal (Fig. 2). The best-corrected visual acuity (BCVA) of the right eye was 0.6. Intraocular pressure was normal, and the results of the dilated fundus examination were unremarkable.

The patient was treated with 0.5% moxifloxacin and 0.1% fluorometholone eye drops four times daily. The patient felt better the next day, and symptoms resolved without sequelae within 5 days. After 1 week, the BCVA in the right eye had improved to 1.0. Medication was halted after 1 week. At the 4-month follow-up, specular microscopy (EM-4000, Tomey, Nagoya, Japan) of the right eye showed a normal pachymetry of 516 μm; thus, indicating a complete resolution of the edema. Cell density (2777 cells/mm^2^) in the right eye was more than that in the left eye (2424/mm^2^). Regarding other parameters, no significant differences were noted. The coefficient of variation was 36% and 36%, and the percentage of hexagonal cells was 44% and 44% in the right and left eye, respectively.

She underwent uneventful cataract surgery in the left eye, which might have decreased the number of endothelial cells; a comparison could not be made, since there were no data on the cell count prior to this episode. We assumed that the endothelial cell density of the left eye prior to this episode was less than that of the right eye, and that snake venom did not induce a serious permanent damage in our patient.

The study protocol conformed to the tenets of the Declaration of Helsinki and was approved by the Ethics Review Committee of Nojima Hospital. Written informed consent was obtained from the patient.

## Discussion

In the English literature, snake venom ophthalmia caused by spitting cobras has been widely reported, whereas only one case of ophthalmia induced by *R. tigrinus* has been documented [1]. Since 1923, 19 cases of ophthalmia caused by *R. tigrinus* were reported in Japan [2, 3]. In these studies, ophthalmic examinations revealed conjunctivitis, keratitis, and corneal edema with Descemet membrane folds [1–3]. Ocular symptoms included eye pain, epiphora, visual disturbance, and flare or cells in the anterior chamber. Some patients have exhibited mydriasis, whereas others have exhibited miosis (Table 1). Ogawa et al. reported a case in which mydriasis was initially observed but miosis was observed over time [2]. In an experimental study on dogs, pupils were miotic at low toxin concentrations but became mydriatic at high concentrations and then became miotic again over time [4]. The difference between pupillary reactions may be attributed to the concentration of the toxin that has entered the eye and time from injury.

**Table 1:** Clinical features and clinical course of venom ophthalmia induced by *Rhabdophis tigrinus*, toad, and Aslepsia spp. (since 1950)

Patient number	Animal, or Plant	Year	Age	Sex	Eye	Pupil	Eye pain	Epiphora	Blurred Vision (BCVA)	Conjuntival injection	Corneal edema	Desmet’s folds	AC Cells	Steroids eye drop, ointment	Systemic steroids	Days to heal
1 [2]	*R.tigrinus*	1953	28	male	L		+	+	+ (0.3)	+	+		+			
2 [2]	*R.tigrinus*	1957	46	female	R	myosis	+	+	+ (0.1)	+	+	+	+	+		3 days
3 [2]	*R.tigrinus*	1959	25	male	L	myosis	+	+	+	+	+	+		+		3 days
4 [2]	*R.tigrinus*	1959	29	male	L	myosis	+		+	+	+	+	+	+		3 days
5 [2]	*R.tigrinus*	1959	45	male	B	mydriasis	+		+	+	+	+		+		5 days
									R(0.3) L(0.3)							
6 [2]	*R.tigrinus*	1959	48	male	B	mydriasis	+		+	+	+	+	+	+		2 days
									R(0.6) L(0.3)							
7 [2]	*R.tigrinus*	1960	36	male	R	myosis	+		+ (0.1)	+	+	+		+		2 days
8 [2]	*R.tigrinus*	1988	33	male	L		+		+ (0.5)	+	+					1 day
9 [2]	*R.tigrinus*	1989	36	male	R		+	+	+	+	+	+				3 days
10 [2]	*R.tigrinus*	1991	39	male	R	myosis	+	+	+ (0.15)	+	+	+	+	+	+	5 days
11 [3]	*R.tigrinus*	2004	67	male	R		+		+ (0.08)	+	+	+	+	+	+	5 days
12 [3]	*R.tigrinus*	2004	60	male	L	myosis		+	+ (0.9)	+	+	+	+	+	+	7 days
13 [3]	*R.tigrinus*	2004	62	male	L				+ (0.6)	+	+	+	+	+	+	5 days
14 [1]	*R.tigrinus*	2014	40	male	R		+		+ (0.1)	+	+	+		+		5 days
15 [Present Study]	*R.tigrinus*	2021	72	female	R		+		+ (0.6)	+	+	+		+		5 days
16 [5]	Toad	2007	31	male	B		+		+	+	+	+		+		3 days
									R(0.4) L(0.5)							
17 [6]	*A.physocarpa*	2017	74	male	B				+	+	+	+		+		6 days
									R(0.2) L(0.2)							
18 [7]	*A.curassavica*	1995	60	male	L				+ (0.3)		+	+				2 days
19 [8]	*A.physocarpa*	2014	65	female	R		+		+ (0.3)	+	+	+		+		7 days
20 [9]	*A.fruticosa*	2011	73	male	B				+	+	+	+		+		2 days
									R(c.f.) L(0.5)							
21 [10]	*A.curassavica*	2019	37	male	R		+		+ (0.3)	+	+	+		+		5 days
22 [11]	*A.Tuberosa*	2017	70	female	L		+		+ (0.05)	+	+	+		+		4 days

*BCVA* Best correlated visual aquity, *A Asclepia*, *R Rhabdophis*

In most cases, topical antibiotics and corticosteroids have been prescribed, sometimes in combination with systemic corticosteroids, systemic antibiotics, antihistamine eye drops, and atropine eye drops. Ocular complications were alleviated in 1–3 days, and the eyes healed within 5–7 days without any sequelae [1–3]. A similar clinical course and good outcome were observed in our patient (Table 1).

Although Duvernoy’s gland toxin of *R. tigrinus* is highly venomous, few deaths have been recorded, which may be because this snake is not ferocious and warlike. In addition, its fangs are short and located at the back of the maxilla, which makes a successful strike on a large target difficult.

Cobras can spit venom from Duvernoy’s glands, where venom is produced and stored, through their fangs at a distance of 1–2 m. Conversely, *R. tigrinus* possesses a series of paired sac-like nuchal glands behind the head. When pressure is applied to the nuchal area, whether internally by the snake’s muscle or the squeeze of an attacker, the thin skin over the nuchal glands can rupture and release secretions over a distance of > 1 m to ward off the attacker [5].

In contrast to the venom in Duvernoy’s glands, the toxin is not synthesized in the nuchal glands of *R. tigrinus*. Instead, this snake consumes poisonous prey (mainly toads), and the toxin is conserved in the nuchal glands. The bufadienolides in the nuchal gland secretion are considered the cause of ophthalmia. The skin of toads contains bufadienolides. Accidental contact of toad toxin with the human eye causes ophthalmia, and its clinical course is similar to that of ophthalmia induced by *R. tigrinus* (Table 1) [6]. The sodium/potassium–adenosine triphosphatase (Na/K-ATPase) pump in the corneal endothelium is known to maintain corneal transparency. Bufadienolides are digitalis-like compounds (DLCs) belonging to a family of steroid hormones. DLCs exhibit digitalis-like effects, including inhibition of the Na/K-ATPase pump, which results in corneal stromal edema and Descemet membrane folds.

We previously reported a case of plant toxin-induced corneal edema due to *Asclepias physocarpa* [12]. The clinical symptoms and course were similar to those described in the present report (Table 1). The plants of the *Asclepias* genus are wildflowers native to tropical Africa and are globally distributed as ornamental plants. Their latex from their stems, leaves, and roots has been shown to contain toxic components called cardenolides. Bufadienolides and cardenolides are similar in structure and function; thus, inhibiting Na^+^/K^+^ ATPase.

Steroid treatment is often administered to reduce ocular inflammatory symptoms. Hatou et al. demonstrated that dexamethasone results in increases in Na/K pump activity in cultured corneal endothelial cells [7]. While the corneal edema can be self-limiting with the clearance of cardenolides or bufadienolides from endothelial cells [8], active anti-inflammatory treatment may be helpful for rapid symptomatic relief [1–3, 9–11, 13].

In conclusion, nuchal glands’ secretion of *R. tigrinu* induces ophthalmia, which resolve spontaneously in a few days. The corneal edema can be self-limiting when inhibition of the toxin has ceased [8], however, the use of topical steroids reportedly increases the activity of Na/K pumps that remain functional; thus, accelerating recovery [1–3, 7, 9–13].

## Data Availability

The data of current case report are available from the corresponding author on reasonable request.

## Abbreviation

*R. tigrinus*: *Rhabdophis tigrinus*
